# Piscivores, Trophic Cascades, and Lake Management

**DOI:** 10.1100/tsw.2002.138

**Published:** 2002-02-05

**Authors:** Ray W. Drenner, Ray K. David Hambright

**Affiliations:** Department of Biology, TCU Box 298930, Texas Christian University, Fort Worth, TX 76129, USA; University of Oklahoma Biological Station, HC-71, Box 205, Kingston, OK 73439, USA, and Israel Oceanographic and Limnological Research, Kinneret Limnological Laboratory, P.O. Box 447, Migdal 14950, Israel

**Keywords:** piscivore, trophic cascade, lake management, food web, fish, plankton, predation, eutrophication, top-down control, bottom-up control

## Abstract

The concept of cascading trophic interactions predicts that an increase in piscivore biomass in lakes will result in decreased planktivorous fish biomass, increased herbivorous zooplankton biomass, and decreased phytoplankton biomass. Though often accepted as a paradigm in the ecological literature and adopted by lake managers as a basis for lake management strategies, the trophic cascading interactions hypothesis has not received the unequivocal support (in the form of rigorous experimental testing) that might be expected of a paradigm. Here we review field experiments and surveys, testing the hypothesis that effects of increasing piscivore biomass will cascade down through the food web yielding a decline in phytoplankton biomass. We found 39 studies in the scientific literature examining piscivore effects on phytoplankton biomass. Of the studies, 22 were confounded by supplemental manipulations (e.g., simultaneous reduction of nutrients or removal of planktivores) and could not be used to assess piscivore effects. Of the 17 nonconfounded studies, most did not find piscivore effects on phytoplankton biomass and therefore did not support the trophic cascading interactions hypothesis. However, the trophic cascading interactions hypothesis also predicts that lake systems containing piscivores will have lower phytoplankton biomass for any given phosphorus concentration. Based on regression analyses of chlorophyll�total phosphorus relationships in the 17 nonconfounded piscivore studies, this aspect of the trophic cascading interactions hypothesis was supported. The slope of the chlorophyll vs. total phosphorus regression was lower in lakes with planktivores and piscivores compared with lakes containing only planktivores but no piscivores. We hypothesize that this slope can be used as an indicator of “functional piscivory” and that communities with extremes of functional piscivory (zero and very high) represent classical 3- and 4-trophic level food webs.

## INTRODUCTION

### Food Webs and Trophic Levels

Understanding food webs is a formidable challenge because food webs in nature may contain hundreds of species, each connected by multiple links of various strengths[1,2,3]. One of the common approaches used to generalize food webs into tractable concepts has been to group organisms into trophic levels, i.e., according to their primary energy source[4,5,6]. For example, producers use solar energy to produce plant tissue; herbivores consume plants; carnivores consume herbivores. Feeding relationships were widely recognized as important sources of interaction within communities, and early ecologists explicitly described regulatory roles for consumers (e.g., Forbes[7]). Nevertheless, trophic structure was generally considered to be a consequence of the amount of energy harvested and transformed by the producers, and each trophic level successively depended on the preceding level as a source of energy[5]. Thus, at least through the first half of the $20^{th}$ century, population regulation was thought to be a function of competition as affected by available resources and climate[8,9,10]. In 1960 this view was challenged by the now seminal paper of Hairston, Smith, and Slobodkin (hereafter HSS)[11], in which they hypothesized that in a 3-level trophic system, herbivore populations were regulated not by available resources, but by consumption by carnivores, while producers and carnivores were resource regulated. During the ensuing 4 decades, numerous extensions of the original 3–trophic level HSS model were developed to include fewer or more trophic levels, as well as energetics (e.g., the exploitation ecosystem hypothesis (EEH))[12,13,14,15,16]. According to the EEH model, even numbers of trophic levels (2 or 4) produce low-standing crops of plants because the herbivore populations flourish. Odd numbers of trophic levels (1 or 3) result in plants controlled by the availability of their resources because herbivores are either absent or suppressed by carnivores.

Though considered a central theory in ecology[17] and included in introductory ecology textbooks[18,19,20], ecologists have debated the utility of HSS and trophic levels in understanding food web dynamics because they do not consider trophic levels to be discretely-defined components of natural food webs[1,3,21,22,23,24,25,26,27,28,29,30]. For example, predators often feed as generalists (or omnivores) and consume prey from more than one trophic level[5,26], thus potentially diffusing predator effects throughout the food web rather than focusing them at particular trophic levels[3]. Moreover, many treatments (theoretical and experimental) of food webs fail to correctly incorporate detritus as a substantial portion of energy and nutrients to consumers[3,25]. Such controversies over the concept of trophic levels and their role in food webs will only be resolved by experimental tests of the various hypotheses[3,26,31,32].

#### Lake Food Webs and the Trophic Cascade

Two of the major food web components of freshwater lakes, plankton and fish, have historically been studied and managed by two separate scientific disciplines: limnology and fisheries biology, respectively[33,34,35,36]. The field of limnology focused on physical, chemical, and planktonic aspects of water quality[37,38,39] and did not consider fish as "driving variables" in lakes. Fish, as well as other organisms, were considered to be "response variables," responding to changes in abiotic and biotic factors. Fish were studied and managed by fisheries biologists to optimize commercial and recreational fisheries yield[40], without consideration of the potential effects of fish assemblages on water quality. This artificial division of components of lake food webs between two separate scientific disciplines delayed our understanding of the effect of fish on food webs and water quality.

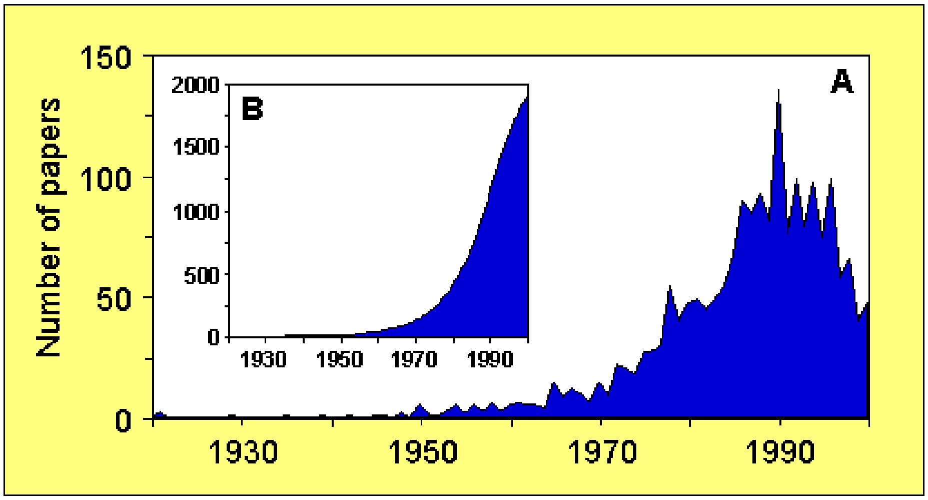

**FIGURE 1.** (A) Number of published scientific articles concerning the effects of fish in lake ecosystems. (B) Inset shows cumulative summary. The database used for this figure can be found at http://www.bio.tcu.edu/drenner/bib.html.

It was not until the pioneering work of Hrbacek et al.[41] and Brooks and Dodson[42] that the field of limnology began to recognize that fish can be important "driving variables" regulating plankton community structure and water quality of lakes. These studies were followed by an increase in research focused on fish effects on food webs of lakes (reviews in Zaret[43], Lazzaro[44], Northcote[45], DeMelo et al.[46], and Brett and Goldman[47]). To date, over 1,900 papers, books, book chapters, and symposia proceedings have been published on the effects of fish in lakes (Fig. 1)[48]. Many of these studies focused on the consumptive effects of predatory planktivorous fish on their herbivorous zooplankton prey. In general, these studies showed that strong regulation of herbivorous zooplankton by planktivorous fish could indirectly cascade (*sensu* Paine[1]) down to the phytoplankton at the bottom of the food chain. These studies of planktivorous fish and their indirect effects on phytoplankton provided sound evidence that the HSS model was not only applicable to 3–trophic level terrestrial systems, but to 3–trophic level pelagic systems as well. Control of herbivorous zooplankton by planktivorous fish allows the phytoplankton to respond to resource availability, which, in lakes affected by high nutrient inputs, results in increased phytoplankton biomass.

Eutrophication is a process in which excessive nutrient loading, especially of phosphorus and nitrogen, leads to phytoplankton blooms and deterioration of water quality[49,50,51]. Because excessive phytoplankton biomass is one of the undesirable symptoms of eutrophication, ecologists hypothesized that piscivorous fish could be used by lake managers to reverse the effects of planktivorous fish on phytoplankton and thereby ameliorate some of the symptoms of eutrophication[38,52]. Although previously examined in only two studies[53,54], this hypothesis became widely accepted in ecology after it was presented as the "concept of cascading trophic interactions" by Carpenter et al.[55] and Carpenter and Kitchell[56]. This concept, also called the *trophic cascade hypothesis,* predicts that a "rise in piscivore biomass brings decreased planktivore biomass, increased herbivore biomass, and decreased phytoplankton biomass"[55] (Fig. 2). Trophic cascades are thus consumer effects in food chains that function in accordance with HSS and EEH models[3,57].

The trophic cascade has become an important paradigm in ecology. Over 600 papers have cited Carpenter et al.'s paper[55] in the 16 years since it was published[58], even though the article contains no original data supporting the trophic cascade hypothesis. The concept of cascading trophic interactions in lakes is now the focus of a book[59] and is included in textbooks on ecology[18,20], limnology[60,61] and fish feeding ecology[62]. Nevertheless, the trophic

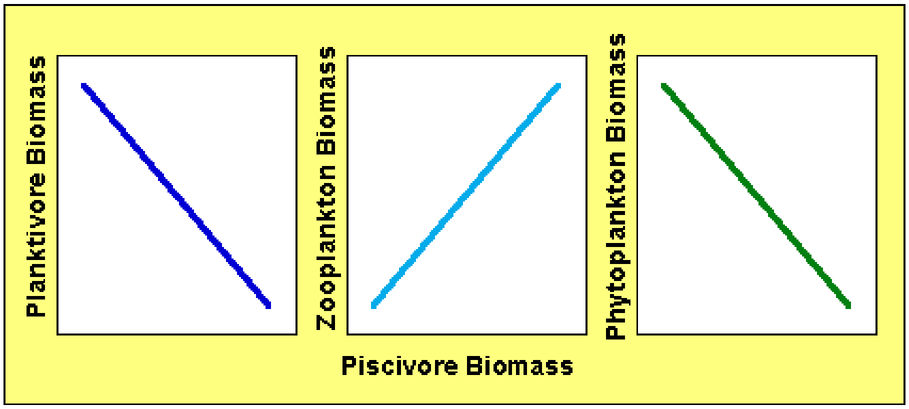

**FIGURE 2**. The trophic cascade hypothesis showing predicted effects of increasing piscivore abundance on planktivorous fish, zooplankton, and phytoplankton. Modified from Carpenter et al.[55].

cascade hypothesis has been characterized as "highly simplistic" because it ignores compensatory responses within the food web that occur following alterations in predator assemblages[63]. Others have offered alternative hypotheses, such as the "bottom-up/top-down" hypothesis, which predicts that top-down effects of piscivores are strong at the top of the food web, but weaken near the bottom, where phytoplankton are regulated primarily by nutrient availability (i.e., from the bottom up)[64]. As stated by Polis and Strong[3], "It is a major challenge to sort out the dynamic forms of aquatic TCs [trophic cascades]: which systems are dominated by TCs and which are not, what other forces come into play, and why."

### APPROACH

Here we review and evaluate the evidence accrued to date pertaining to piscivory and the trophic cascade hypothesis in lakes, reservoirs, and ponds. We searched the scientific literature for experimental studies and surveys that examined the potential for piscivore effects on planktivorous fish to cascade through the herbivorous zooplankton to phytoplankton. We did not include studies that evaluated piscivore effects on planktivorous fish and zooplankton but not phytoplankton[65] or studies in systems in which planktivore reproduction was restricted[66]. We also did not include Lake Michigan, which was thought to exemplify the beneficial biomanipulation effects of piscivore stocking[67] until additional analyses suggested that a suite of nutrient-based processes were responsible for observed changes in the algal communities[68,69,70,71,72].

We found 33 experiments and 6 surveys examining piscivore effects on phytoplankton biomass and water quality (Table 1). We divided the experiments into four categories: piscivore addition[54,57,73,74,75,76,77,78,79,80,81], piscivore removal[79,80,81], piscivore enhancement[76,82,83,84,85,86,87,88,89,90,91,92,93,94,95,96,97,98,99,100,101,102], and community replacement[41,84,90,99,100,103,104,105,106,107,108,109,110,111,112,113,114]. Many of these experiments were conducted as biomanipulation studies and accompanied by supplemental manipulations (i.e., nutrient reduction, partial fish removal, fish assemblage elimination, fish addition) with the objective of improving water quality, not assessing piscivore effects. Although these studies yield valuable information about biomanipulation and its effectiveness as a lake management strategy[82,115,116,117,118,119,120,121,122], they are confounded[123] because the piscivore effects are inseparable from the effects of the supplemental manipulations and thus cannot be assessed.

**TABLE 1**
**Experimental Studies and Field Surveys Examining the Effects of Manipulations of Populations of Piscivorous Fish on Phytoplankton Biomass**

| Site | Design | Piscivore Manipulation | Confounding Factors[a] | Algal Biomass Decline |
|---|---|---|---|---|
| Balancing Reservoir[73,74] | Expt. | Addition | None | No |
| Cornell Univ. Ponds[57] | Expt. | Addition | None | Yes |
| Grafenhain Exptl. Water[54,75,76] | Expt. | Addition | None | Yes |
| Kansas Univ. Ponds[77] | Expt. | Addition | None | No |
| Lake 221[78] | Expt. | Addition | None | No |
| Mouse Lake[79,80] | Expt. | Addition | None | No |
| Tuesday Lake[81] | Expt. | Addition | PFR | --- |
| Peter Lake[81] | Expt. | Removal | FA | --- |
| Ranger Lake[79,80] | Expt. | Removal | None | No |
| Bautzen Reservoir[76,82] | Expt. | Enhancement | None | No |
| Lake Bleiswijkse Zoom[83,84,85] | Expt. | Enhancement | PFR | --- |
| Lake Breukeleveen[86] | Expt. | Enhancement | PFR | --- |
| Feldberger Hausee[87] | Expt. | Enhancement | PFR | --- |
| Fredericksborg Castle Lake[88,89] | Expt. | Enhancement | PFR | --- |
| Lake Gjersjon[90,91] | Expt. | Enhancement | None | Yes |
| Johnson Bass Pond[92] | Expt. | Enhancement | PFR | --- |
| Lake Klein Vogelenzang[93] | Expt. | Enhancement | PFR, NR | --- |
| Lake Sovdeborg-II[94] | Expt. | Enhancement | None | No |
| Lingese Reservoir[95] | Expt. | Enhancement | NR | --- |
| Lake Lyng[96,97] | Expt. | Enhancement | None | Yes |
| Rimov Reservoir[98] | Expt. | Enhancement | PFR | --- |
| Lake Wirbel-Phase I[99,100] | Expt. | Enhancement | PFR | --- |
| Lake Wolderwijd[101,102] | Expt. | Enhancement | PFR | --- |
| Lake Christina[103,104,105] | Expt. | Com. Replace. | FAE | --- |
| Delavan Lake[106] | Expt. | Com. Replace. | FAE | --- |
| Lake Haugatjern[107] | Expt. | Com. Replace. | FAE | --- |
| Lake Ijzeren Man[108] | Expt. | Com. Replace. | FAE | --- |
| Maltanski Reservoir[109] | Expt. | Com. Replace. | FAE | --- |
| Lake Mosvatn[90,110] | Expt. | Com. Replace. | FAE | --- |
| Poltruba Backwater[41] | Expt. | Com. Replace. | FAE | --- |
| Round Lake[111] | Expt. | Com. Replace. | FAE | --- |
| Lake Wirbel-Phase II[99,100] | Expt. | Com. Replace. | FAE, PFR | --- |
| Zwemlust[84,112,113,114] | Expt. | Com. Replace. | FAE | --- |
| Argentinian Lakes & Res.[124] | Survey | | | Yes |
| Lake St. George[125] | Survey | | | No |
| Michigan Ponds[53] | Survey | | | Yes |
| Ontario Lakes[126] | Survey | | | No |
| Peter and West[127,128] | Survey | | | Yes |
| Swedish Lakes[129] | Survey | | | No |

[a] PFR = partial fish removal; FA = fish addition; NR = nutrient reduction; FAE = fish assemblage elimination.

Following removal of all studies for which we had sufficient information to assess confounding, we were left with 11 experiments (6 piscivore additions[54,57,73,74,75,76, 77,78,79,80], 1 piscivore removal[79,80], and 4 piscivore enhancements[76,82,90,91,94,96,97]) and 6 surveys[53,124,125,126,127,128,129] (Table 1). Of the experiments, 4 (2 piscivore additions and 2 piscivore enhancements) detected reductions of phytoplankton biomass due to piscivore effects. However, 7 experiments (4 piscivore additions, 1 piscivore removal, and 2 piscivore enhancements) failed to document any piscivore-mediated effects on phytoplankton biomass. Among the surveys, 3 studies found piscivore effects on phytoplankton biomass and 3 did not. Thus, the majority of the nonconfounded studies (10 out of 17) failed to support the trophic cascade hypothesis.

In the above summary, we used a technique called *vote counting* that consists of tallying all studies that either detected or failed to detect piscivore effects on phytoplankton in accordance with predictions of the trophic cascade hypothesis. Although this technique is widely used in summarizing literature data in ecology, it perpetuates type II statistical errors (failing to detect a true effect) in the original studies, particularly those with small sample size and high variability[47]. Therefore, to examine piscivore effects on phytoplankton biomass, we also took an alternative approach in which we examined the relationship between chlorophyll (a proxy for phytoplankton biomass — but see Smith[51]) and total phosphorus in systems with and without piscivores. Several ecologists have predicted that lakes with piscivores will have lower primary productivity or lower chlorophyll for a given amount of phosphorus compared with lakes without piscivores[55,56,128,130,131,132,133,134] (Fig. 3). To test this prediction, we constructed chlorophyll–total phosphorus relationships for systems with and without piscivores using data from the 13 (of the total 17) nonconfounded experiments and surveys that presented phytoplankton biomass as summer chlorophyll (Table 1). Three studies (Grafenhain Exptl. Water[54,75,76], Bautzen Reservoir[76,82], and Lake 221[78]) presented algal biomass as wet weight or carbon and one (Kansas Univ. ponds)[77] used unitless fluorescence and therefore cannot be used in our comparison. For studies in which chlorophyll and total phosphorus data were presented in graphical form, we scanned and digitized the data from the figures.

As would be expected from a diverse set of studies, the observation periods for chlorophyll and total phosphorus were not consistent across the studies, e.g., some systems were sampled once in the summer while others were monitored multiple times during a summer. Regardless, we estimated mean summer chlorophyll and total phosphorus values for each summer for each system (i.e., for each lake-summer). For the 13 nonconfounded studies, there were 42 and 55 lake-summers for planktivore and piscivore systems, respectively. Treating each lake-summer from an ecosystem with multiple years of data individually slightly inflates the degrees of freedom in statistical comparisons but is preferred because averaging across years hides the natural residual variation in algal biomass[135].

The slope of the chlorophyll–total phosphorus relationship was three times higher in planktivore systems compared with piscivore systems (Figs. 4A and B). Hence, as predicted by the trophic cascade hypothesis, we found a general pattern of lower chlorophyll concentrations per phosphorus concentrations in the systems containing piscivores relative to the systems with only planktivores. This pattern is not readily detectable for systems with relatively low total phosphorus concentrations (i.e., less than 25 µg/l), but in systems with total phosphorus concentrations greater than 100 µg/l, the difference in chlorophyll levels across the two types of systems is substantial.

We compared our chlorophyll–total phosphorus regressions with regressions from lakes with various types of fish communities. The chlorophyll–total phosphorus regression in lakes without piscivores (i.e., planktivore lakes) had a slope similar to data for 3-link systems shown by Mazumder[135] for experimental systems ranging across small mesocosms to large lakes and reservoirs, and by Quiros[136] for Argentine lakes and reservoirs without piscivores (Fig. 5A). The piscivore regression is also similar to that shown for Argentine lakes and reservoirs containing piscivores[136]. For unmanipulated Canadian lakes both with and without piscivores, Currie et al.[126] concluded that there was no difference in the chlorophyll–total phosphorus relationships across systems with and without piscivores. Their combined regression lies intermediate to the two sets of regressions shown in Fig. 5A.

We also compared our chlorophyll–total phosphorus regressions with some of the more commonly cited chlorophyll–total phosphorus relationships[138,139] (Fig. 5B). Like the regression of Currie et al.[126], these relationships fell intermediate between the regressions for planktivore and piscivore lakes. Although we do not have information regarding fish assemblages in the systems incorporated into those relationships, their intermediate placement suggests that a portion of the lakes contained piscivores. This is consistent with the worldwide practice of stocking piscivorous fishes for recreational and commercial fishing[140].

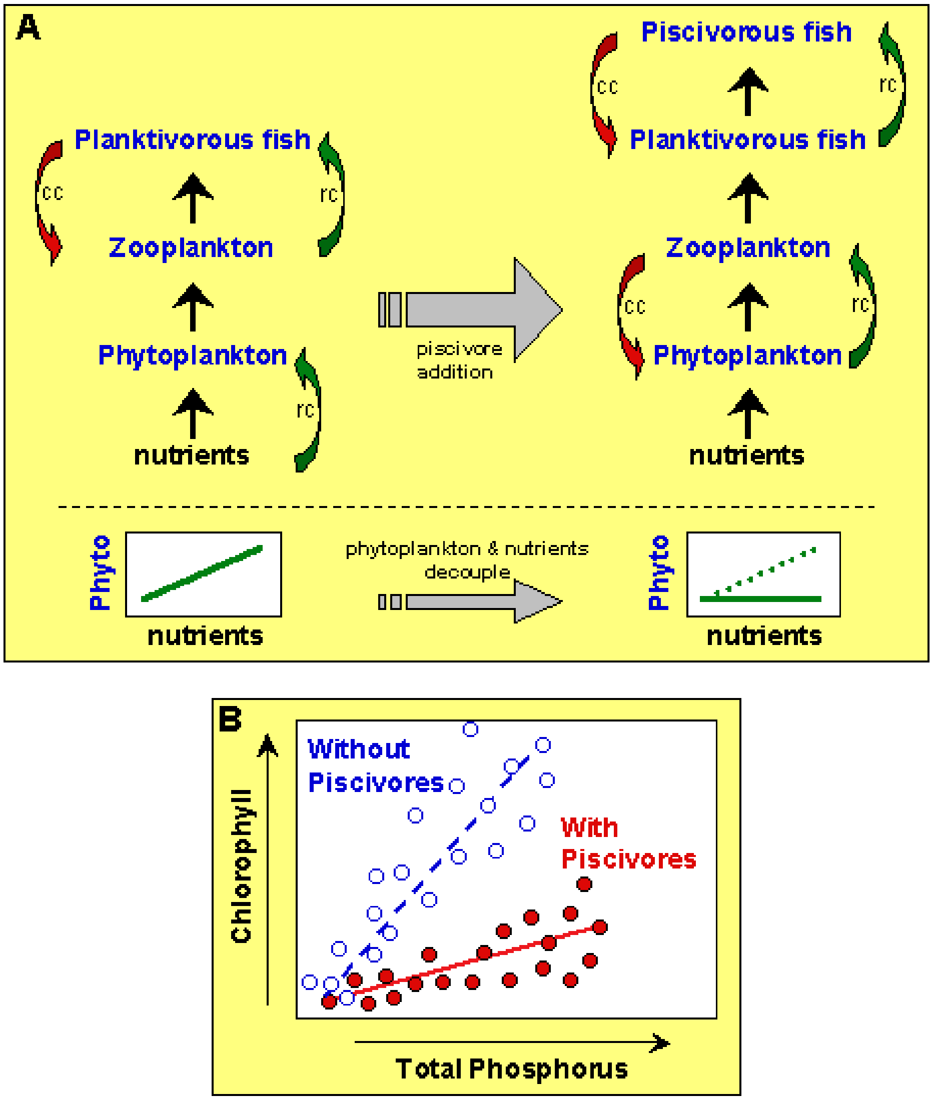

**FIGURE 3.** (A) Trophic cascade hypothesis viewed according to HSS[11]. The food chain on the left functions predominantly as a 3-level system. The food chain on the right results from the addition of piscivores and functions predominantly as 4-level system. **Red** arrows denote consumer control (cc) and **green** arrows denote resource control (rc). Note that the shift from a 3-level food chain to 4-level food chain results in a decoupling of phytoplankton from nutrients (e.g., phosphorus). (B) Hypothetical effect of piscivores on chlorophyll–total phosphorus relationship in lakes with planktivorous fish.

### DISCUSSION

#### Why Is Phytoplankton Biomass Lower in the Presence of Piscivores?

Predation by piscivores can dramatically reduce densities and biomass of planktivorous fish, especially small young-of-year fish or adult fish of small-bodied, minnow-like species with soft fin rays[53,57,74,128,141,142,143,144,145,146,147]. Indeed, in some of the studies shown in Table 1 that found piscivore effects on phytoplankton, the planktivore assemblages were comprised of high-vulnerability species. For example, the two piscivore addition experiments that found piscivore cascading effects on phytoplankton were conducted in systems in which planktivory

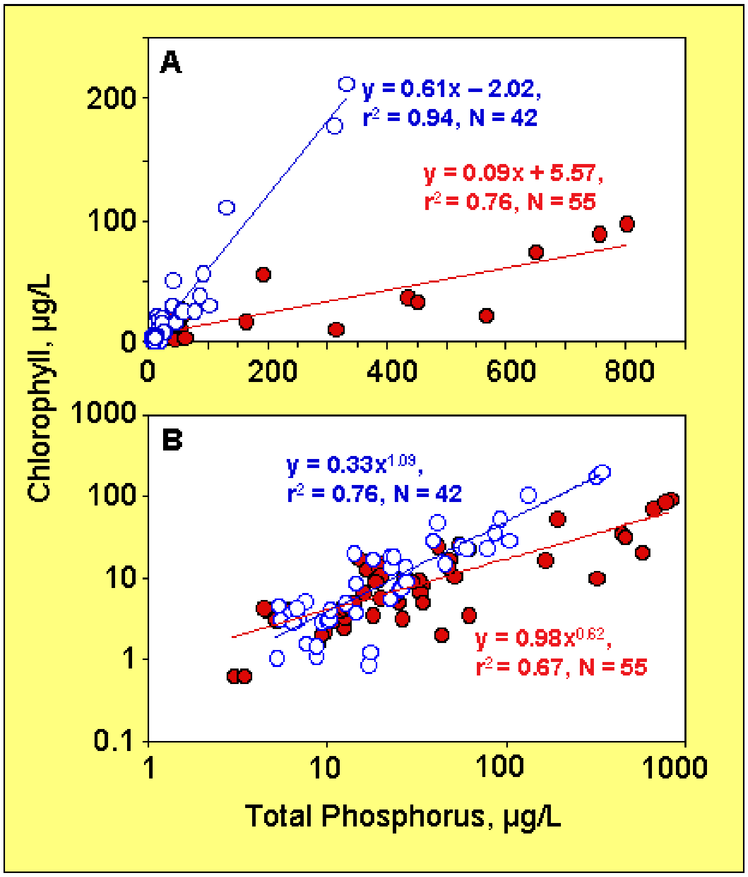

**FIGURE 4.** Chlorophyll–total phosphorus relationships in systems containing planktivorous fish with (**red, closed**) and without (**blue, open**) piscivorous fish. Each point represents a lake-summer from the nonconfounded experiments and surveys shown in Table 1.

was dominated by small, highly vulnerable minnow species (fathead minnows[57]; sun bleak[54,75,76]). The three surveys consistent with the trophic cascade hypothesis included systems in which the planktivore assemblages were also dominated by small, highly vulnerable species (various atherinid spp.[124]; minnows[53, 127,128]). In addition to absolute reductions in planktivore abundance, piscivores may elicit behavioral responses in planktivores that result in reductions in planktivory even though fish densities are not reduced. For example, planktivores, responding to visual and/or chemical cues from piscivores[148], may leave the pelagic zone and seek refuge in the littoral zone[91,143,149,150,151,152,153,154]. Most studies examining the trophic cascade did not quantify habitat use by planktivores, so it is not possible to infer the importance of this indirect mechanism of reduction of pelagic planktivory in affecting the piscivore-mediated trophic cascades. Nevertheless, reduced densities of planktivorous fish in the pelagic zone, whether due to consumptive or behavioral mechanisms, results in lower predation pressure on pelagic zooplankton.

Because most planktivores are size-selective predators, reduced predation pressure typically results in increased abundances of large zooplankton[44,45]. Larger zooplankton can exert greater grazing pressure on phytoplankton compared with smaller zooplankton[155,156,157,158,159]. Thus, the key trophic cascade–related effect on zooplankton may not be an increase in zooplankton biomass but an increase in zooplankton size[52,134,160] (but see Tessier et al.[161]). Support for this concept comes from analysis of relationships between chlorophyll and total phosphorus among oligotrophic and eutrophic temperate lakes, revealing that systems

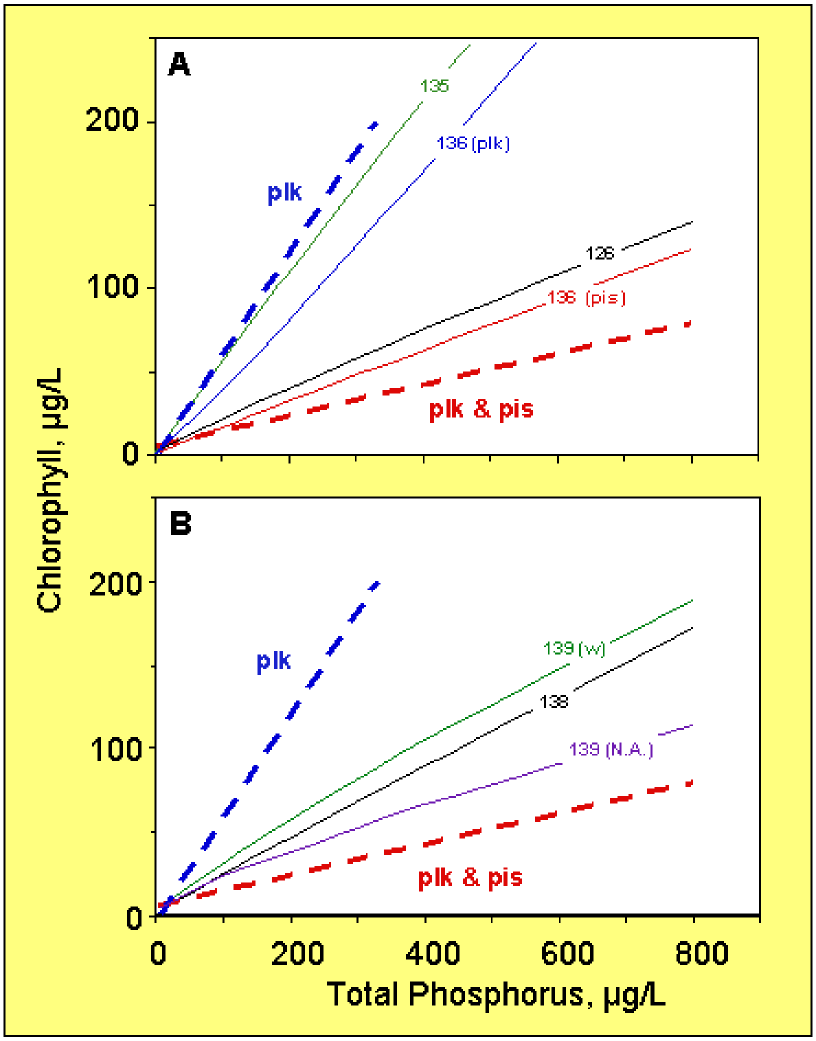

**FIGURE 5.** Comparison of chlorophyll–total phosphorus relationship in systems containing planktivorous fish with (**red** dashed line) and without (**blue** dashed line) piscivorous fish (from Fig. 4) to previously published chlorophyll–total phosphorus relationships. The numbers on the graph lines denote the literature reference number. (A) The **green** line is from Mazumder[135] for systems containing only planktivorous fish. The black line is from Currie et al.[126] for systems with and without piscivores. The remaining lines are from Quiros[136] for Argentine lakes and reservoirs containing only planktivorous fish (**blue** line) and both planktivorous and piscivorous fish (**red** line). (B) **Green** and **purple** lines are from Nurnberg[139] for World and North American lakes and reservoirs; the black line is from OECD[138]. Note that all regression lines have been extended to the range for data shown in Fig. 4.

having large *Daphnia* had lower chlorophyll per total phosphorus than those with small *Daphnia*[135].

Although most studies of the trophic cascade effects of piscivores focus on pelagic food webs (Fig. 2), there are often complex interactions between pelagic and littoral zones that can contribute to the pelagic phytoplankton responses to piscivores[162,163,164,165]. For example, the predominant planktivores in many lakes are young benthivorous fish, adults of which feed primarily on invertebrates on the lake bottom. Benthic feeding fishes commonly are associated with high turbidity due to sediment resuspension during feeding and little macrophytic vegetation due to low light penetration to the lake bottom and uprooting. Reductions of these facultative planktivores by piscivores can lead to increased macrophytic vegetation. Proliferation of macrophytes can indirectly lead to reduced phytoplankton abundances because macrophytes reduce turbulence, take up nutrients from the water, and provide a refuge for zooplankton against fish predation. Piscivore reductions of facultative planktivores can act as a trigger for switching a lake from a highly turbid, dense phytoplankton–dominated state into a clear, macrophytic vegetation–dominated state, even in systems with high nutrient loading. Though macrophytes are commonly enhanced in biomanipulation studies[122], macrophytes were typically not reported in the nonconfounded experiments and surveys reviewed here. The one exception is that Spencer and King[53] noted qualitatively increased macrophyte abundance in ponds containing piscivores. However, several of the studies supporting the trophic cascade hypothesis were conducted in relatively small, shallow systems (0.1-ha experimental ponds[57]; 3.4-ha lake[127]; 9.9-ha lake[96,97]) in which the littoral zone (and hence, macrophytes) may have played an important role in the phytoplankton response to piscivory in these systems. In shallow lakes, long-term reductions of phytoplankton associated with piscivore manipulations may be maintained by the macrophytic vegetation and its effect on nutrient cycles and turbidity[163,164,165].

An additional indirect pathway for piscivore reduction of phytoplankton can involve the reduction of nutrients transported from the lake bottom to the water column by benthic-feeding planktivores. Though often classified as "pelagic" planktivores, many of these fishes can obtain up to 50–90% of their energy from benthic sources[166,167]. For example, Schindler et al.[134] reported that planktonic organisms accounted for less than 30% (by biomass) of the diets of planktivorous minnows, mud minnows, and perch; the bulk of their diet was from littoral sources (30% benthic insects, 40% periphyton). Planktivorous fish can transfer nutrients from the benthic zone to the pelagic zone when they consume benthos or detritus and excrete the phosphorus and nitrogen into the water column[134,167,168,169,170,171,172,173,174,175,176,177,178,179,180,181]. Also, the physical activity of fish foraging in the sediments (i.e., bioturbation) can transfer substantial amounts of dissolved and particulate nutrients from the sediments into the water[182]. In lakes where such nutrient pumping effects and benthic feeding activity of benthivorous fish contributes significant amounts of nutrients for phytoplankton growth, suppression of populations of facultative planktivores by piscivores also reduces the transfer of nutrients from the benthic zone to the pelagic zone. Several studies supporting the trophic cascade hypothesis contained abundant benthic-feeding planktivores (roach, pumpkinseed sunfish[57,90,96]), though nutrient transport from the benthic and littoral areas by these fishes was not quantified.

### Why Is Phytoplankton Biomass Not Affected by the Presence of Piscivores?

Perhaps one of the most appealing aspects of the trophic cascade hypothesis is its apparent simplicity. Lotka-Volterra oscillations between predator and prey densities are among the first topics encountered by students of ecology, and even today it is difficult for students to understand that the concepts espoused in HSS were treated as novel ideas during the 1960s. So why is the trophic cascade hypothesis, such a simple and straightforward concept, not supported by experimental tests? As with the original concept, the answer also seems obvious: natural communities are not comprised of simple linear food chains and every level of a food chain contains species that have various morphological, behavioral, and chemical defenses to deter consumers. Indeed, this has been one of the central arguments against HSS and other models that involve discrete trophic levels[3]. Each trophic level is comprised of many different species, each with differing food preferences and vulnerabilities to predators, such that the net effect is a blurring of the concept of trophic level and a dampening of the effects of piscivores on phytoplankton biomass. For example, large deep-bodied prey fish are less vulnerable to piscivory because piscivores are gape-limited predators and can only consume prey fish having body depths less than mouth gape[183,184,185,186,187,188,189,190,191,192,193]. Therefore, instead of planktivores being greatly reduced by piscivores[53,76], intense piscivory can shift the planktivore assemblage from one of small-bodied, highly vulnerable individuals to one of larger-bodied, less vulnerable individuals[77,82,188]. This mechanism could be involved in the lack of support for the trophic cascade hypothesis in the studies reviewed here. For example, in contrast to studies supporting the trophic cascade hypothesis, the planktivore assemblages in the nonsupporting experiments in Table 1 were dominated by relatively large, deep-bodied, invulnerable taxa such as centrarchids and perch[77,78,79,80]. Similarly, relatively large-bodied invulnerable planktivores (centrarchids, perch, coregonids) were present in the survey studies that failed to support the trophic cascade hypothesis[125,126,129]. A shift in fish community structure under increased piscivory is explained by the interaction of competition and predation. In 3-link systems, planktivorous fish are mainly controlled by competition and relatively small-bodied species tend to fare better under resource-limitation; but in 4-link systems in which planktivorous fish assemblages are expected to be predator-limited, large-bodied taxa have the advantage[39]. Bronmark and Miner[189] even found that fish body morphology within a species could also respond to presence of piscivores, with carp body depths increasing in the presence of piscivorous pike.

Compensatory shifts at the planktivore level are not restricted to planktivorous fish. There are many relatively large invertebrate planktivores (such as larvae of the phantom midge *Chaoborus,* the opossum shrimp *Mysis,* and even large cladoceran zooplankton, *Leptodora* and *Polyphemus*) that can inflict relatively high mortality on zooplankton populations[194,195,196]. However, in the presence of planktivorous fish, populations of invertebrate planktivores are also typically suppressed or individuals are restricted in habitat use so as to be ineffective in population regulation of zooplankton assemblages. When planktivorous fish populations are suppressed by piscivores, these invertebrate planktivores increase and suppress the large herbivorous zooplankton. Such a response to increased piscivory was documented in two of the experimental tests of the trophic cascade hypothesis[76,197,198]. In both cases, increased planktivory by *Chaoborus* cancelled many of the effects of decreased planktivorous fish abundance following the addition or enhancement of piscivore populations.

A further complication arises from the fact that most piscivorous fish are actually generalists whose diets not only include fish but also invertebrates. Piscivores, such as largemouth bass, zander, pike, and perch, undergo ontogenetic diet shifts from zooplanktivory and benthivory as young of year and juveniles to facultative piscivory as adults[199,200,201,202,203,204,205]. Therefore, instead of enhancing zooplankton via the trophic cascade, the addition of piscivorous fish to lakes may actually lead to increased predation pressure on zooplankton, particularly in years of high reproductive success[206].

At the herbivorous zooplankton level, small-bodied zooplankton are not easily located by visually feeding planktivorous fish, and evasive zooplankton such as copepods are able to avoid capture by filter-feeding fish[44,45]. Zooplankton can also alter their vulnerability to planktivorous fish by vertically migrating and residing in the deep (and therefore darker) layer of the water column during the day to avoid visually feeding fish and returning to the surface layer during the night to feed[207,208,209,210,211,212,213,214]. Zooplankton that vertically migrate are not subjected to intense predation pressure from planktivores and may not respond according to predictions of the trophic cascading interactions hypothesis.

Phytoplankton also have morphological and chemical defenses to lessen the effects of grazing. Large filamentous and colonial phytoplankton are not consumed efficiently by filter-feeding zooplankton[156,215,216,217,218,219] (but see Schoenberg and Carlson[220]). Several studies have shown dramatic shifts in phytoplankton composition following increases in grazing pressure resulting from piscivore enhancement. For example, in one of the earliest attempts at biomanipulation, Shapiro and Wright[111] found that enhancement of *Daphnia* populations led to a condition of phytoplankton dominance by large, filamentous cyanobacteria. Since then, other studies have documented increases in relative abundance of large inedible algae following enhancement of herbivorous zooplankton assemblages[74,76]. Physical edibility is not the only mechanism preventing zooplankton from effectively controlling phytoplankton populations. Some phytoplankton are toxic to herbivorous zooplankton, including cyanobacteria that are the most widely spread and problematic freshwater nuisance algal taxa in eutrophic lakes[221]. Thus, even if piscivore effects cascade through the planktivores and result in enhanced zooplankton populations, the intensified grazing can produce a shift in the relative abundances of various phytoplankton species, such that inedible phytoplankton like large cyanobacteria increase in abundance and compensate for declines in edible phytoplankton[74,76].

In summary, of the 17 nonconfounded experimental studies and surveys, 7 found evidence of a trophic cascade from piscivores to phytoplankton and 10 did not. Several factors may have contributed (both independently and in conjunction) to these outcomes, including differential planktivore vulnerabilities and behaviors, shifts in zooplankton body size, changes in macrophyte abundances, littoral and benthic nutrient transfer to pelagic waters, and phytoplankton edibility. Without further experimentation and quantification of these contributing factors, is not possible to identify which of these mechanisms were involved in the phytoplankton response. As such, we are left to conclude that it is clear that trophic cascades from piscivores to phytoplankton can occur but it is not clear when and in which systems they will occur.

### Management Implications

Because cultural eutrophication is caused by excessive nutrient loading, the principal form of eutrophication abatement has historically been reduction of nutrient input[49,50,51,116]. The "possibility of restructuring the biological community of lakes as a direct approach to combating eutrophication" was first emphasized by Shapiro et al.[52], who proposed the concept of biomanipulation as "a series of manipulations of the biota of lakes and of their habitats to facilitate certain interactions and results which we as lake users consider beneficial — namely reduction of algal biomass and, in particular, of blue-green species." To most lake managers, though, the use of the term *biomanipulation* is restricted to top-down control of the food web via partial to complete removal of planktivorous fishes using artificial (poison, harvest) or natural (piscivory) means[116,117,118,119,120,121,122,222,223].

Some have postulated that biomanipulation has many advantages over nutrient control as an approach to controlling eutrophication. Conventional methods of controlling eutrophication, such as sewage treatment and diversion, are management-intensive and expensive. Moreover, nutrient reduction can also reduce primary production, and, ultimately, fish production and sport fishing[224,225,226,227,228]. Biomanipulation is assumed to be less expensive and can be applied to lakes with nutrient input from nonpoint sources, though it is still likely to be management intensive[121]. Biomanipulation is also compatible with sport fishing when it involves enhancement of piscivorous game fish populations, but overharvest of piscivores by anglers must be regulated. As Ney[229] stated, "Top-down management appeals to everybody. If you want cleaner water, stock more game fish; anglers, swimmers, and boaters will all be happy."

Although some ecologists have been skeptical about whether biomanipulation of the food web will be stable and offer a long-term solution to eutrophication-related water quality problems[12,46,63,76,118,223,230], biomanipulation has been applied in many North American and European lakes[122]. Many biomanipulation strategies involve piscivore stocking, either as an addition to a previously piscivore-free system, as supplemental enhancement of existing piscivore assemblages, or as part of a complete fish assemblage replacement following elimination of fish by poisoning or draining (Table 1). Of all the forms of biomanipulation commonly applied, stocking of piscivores with no additional manipulation seems to be the least effective at achieving reductions in phytoplankton biomass, though when coupled to supplemental planktivore harvests, piscivore enhancements show more promise[122].

One criticism of using piscivores to biomanipulate lakes has been that piscivores may have strong effects on their fish prey, but at the bottom of aquatic food webs, algal communities are most strongly influenced by resource availability[47,64]. Therefore, trophic cascades were expected to be decoupled at the zooplankton-phytoplankton linkage[46,231], especially in eutrophic lakes which can be dominated by cyanobacteria that are resistant to grazing by herbivorous zooplankton. However, others have predicted that the effects of piscivores on phytoplankton may be greatest in eutrophic lakes[128,133,134,231]. Though not basing their conclusions directly on biomanipulation or trophic cascade studies involving piscivores, Carney[232] and Elser and Goldman[233] concluded that food web control should be most effective at intermediate levels of trophy (i.e., in mesotrophic lakes).

In a recent review, Benndorf et al.[223] concluded that biomanipulation success is dependent on phosphorus levels. They suggested that most biomanipulations that failed did so because they were attempted either in deep eutrophic to hypertrophic lakes in which extremely high phosphorus loading prevented any negative responses in the phytoplankton, or in oligotrophic and ultra-oligotrophic lakes in which phosphorus is so low that there is "no possibility" or "no need" to exert top-down control. Our analysis does not support the first conclusion of Benndorf et al.[223] relating to eutrophic and hypertrophic lakes. Although our chlorophyll–total phosphorus regression analysis (see Fig. 4) excludes a majority of biomanipulation studies due to issues of confounding effects, it also indicates a role for phosphorus in the outcome of piscivore manipulations. Rather than an absolute reduction in phytoplankton biomass, our analysis suggests that the amount of phytoplankton biomass relative to the amount of phosphorus should be lower in systems containing piscivores and that the difference between chlorophyll–total phosphorus ratios in 3-link and 4-link systems is expected to be greatest in high-phosphorus, eutrophic lakes. In contrast, and in agreement with Benndorf et al.'s[223] second conclusion relating to oligotrophic systems, we suggest that there is little chance for piscivore manipulations to reduce phytoplankton in oligotrophic lakes. More correctly stated, we suggest that there is little chance for "detecting" any piscivore effects in these oligotrophic lakes. We concur with Benndorf et al.[223] that there is simply no need for piscivore manipulations in oligotrophic lakes because phytoplankton biomass is already so low in these low-nutrient systems. We would argue that many "failed" piscivore manipulations have done so because they were attempted in low-nutrient lakes, in which chlorophyll and total phosphorus were already very low. (Note that most points in Fig. 4 — 58 out of 97 total — are below chlorophyll of 10 µg/l and total phosphorus of 25 µg/l.) Thus, not only is the need for biomanipulation greater in eutrophic lakes, so too are the chances for detecting effects of piscivores greater with increased eutrophication, at least over the range of total phosphorus concentrations examined in Fig. 4.

Beyond lake phosphorus content, there are other complicating issues that may impinge upon the success of piscivores having trophic cascading effects on phytoplankton. For example, lake size may play an important role, not only due to its influence on the establishment of macrophytic vegetation and subsequent switch to an alternate stable state, but also due to relationships between lake size and species diversity. Fish community complexity and number of fish species increase as a function of lake surface area[235,236,237,238,239], and only very small lakes will have planktivore communities without piscivores. An exception may be tropical lakes that do not contain piscivores, but these lakes have many other attributes that will prevent biomanipulation from being successful, including: invertebrate predation is often strong and may prevent increases in the abundance of large herbivores; and herbivores such *Daphnia* tend to be smaller and less abundant, and the planktivore community is dominated by filter-feeding omnivorous fish (clupeids, cyprinids, cichlids) that rapidly grow to sizes too large to be consumed by piscivores[240]. Moreover, stocking of exotic piscivores into tropical lakes could have undesirable effects on the fish community as observed in Lake Gatun[241] and Lake Victoria[242,243,244,245,246,247], where species became extinct.

Our confirmation of a strong effect of piscivores on chlorophyll–total phosphorus relationships suggests that food web structure may in part be responsible for the strong interlake variability in the response of algal biomass to changes in total phosphorus during lake restoration that was reported by Smith and Shapiro[248]. We predict that algal biomass may progressively be less sensitive to a given degree of nutrient removal as the proportion of piscivores (relative to total fish biomass) increases.

### CONCLUSIONS

It has been widely assumed outside aquatic ecology that the theory of trophic cascades has been rigorously tested in aquatic systems and a consensus is emerging on the magnitude and frequency of cascading trophic effects in aquatic communities[30,249,250]. While it is true that great support exists for cascading effects of planktivorous fish to phytoplankton, via strong effects on the zooplankton[44,45,47], there have been remarkably few tests of piscivores and their cascading effects on phytoplankton. Of the 1,900 references on fish effects (Fig. 1), 39 involve piscivore effects on phytoplankton, but only 17 of these studies do so without confounding effects. These experiments and surveys show that the effects of piscivores can cascade down through the food web and alter the species composition at all levels of the pelagic food web but usually fail to reduce phytoplankton biomass.

Our analyses suggest that planktivore lakes have more chlorophyll per unit total phosphorus than piscivore lakes, but these relationships need to be treated as a hypothesis that needs more study. We further hypothesize that the slope of the chlorophyll–total phosphorus regression can be used as an indicator of “functional piscivory” (Fig. 6) and that the extremes of functional piscivory (zero and very high) represent the 3- and 4-trophic level systems described in previous food web models. Functional piscivory would not just be a function of piscivore density, but would also include variables such as piscivore species and behavior relative to the prey fish assemblage[251]. We were unable to test these hypotheses rigorously at this time because published fisheries studies of the relationship of fish biomass and production, nutrients, and chlorophyll do not report fish species composition[224,225] and studies of fish removals do not report any water quality changes before and after the change in fish populations[252]. Unfortunately the fields of limnology and fisheries are still paying the price for the historic partitioning of the ecosystem between two scientific disciplines.

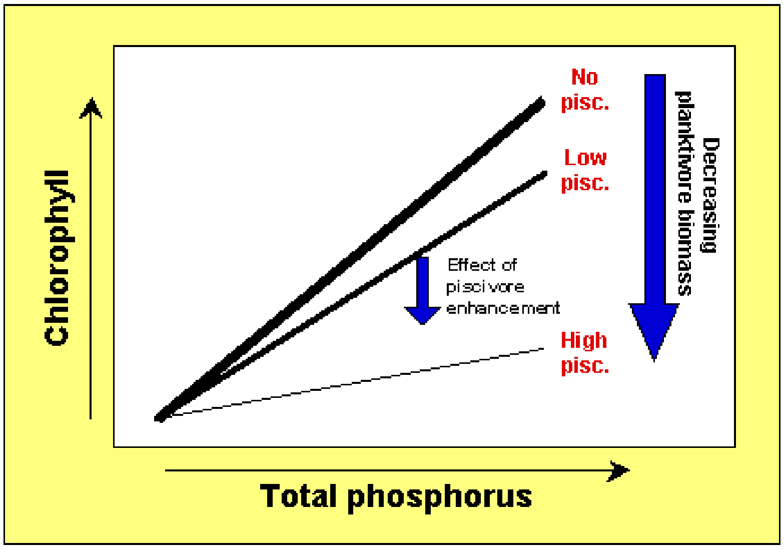

**FIGURE 6.** Theoretical effect of piscivores on the chlorophyll–total phosphorus relationship in lakes. The heavy line indicates the relationship predicted in lakes that contain planktivorous fish but no piscivorous fish and therefore function as 3–trophic level systems. The thin line indicates the relationship predicted in lakes that contain abundant piscivorous fish and therefore function as 4–trophic level systems. The intermediate line indicates the relationship predicted in lakes that contain low populations of piscivorous fish and therefore function more like 3–trophic level systems.

Early in the debate about consumer control of trophic levels, Hairston and Smith[31] stated, "It is clear that observation and experimentation, rather than argument, will eventually resolve the question." However, after several decades of experimentation and study, the controversy still lingers[3,6,253]. Like other fields of science[254], ecology progresses by testing and rejecting hypotheses, either through experiments or field surveys. Most of the experiments on piscivores and the trophic cascade have been with very small ponds and lakes with only a few fish species, while surveys typically encompass larger, more complex systems. Because community responses can be scale dependent[255,256,257], differences in results of experiments and surveys might be expected. This may explain, in part, why studies examining the trophic cascade hypothesis have produced such seemingly inconsistent results. Meta-analysis overcomes this to some degree, integrating community responses across a wide range of ecological variables not found in individual experiments. Our analysis (presented in Fig. 4 and Fig. 5) suggests that piscivores may affect phytoplankton–total phosphorus relationships, but it yields no insight into how individual systems may respond to piscivores. Future experiments examining trophic cascades must focus on species-specific dynamic traits such as behavior, morphology, and body size that are key elements affecting the outcomes of predator-prey interactions[251]. Moreover, if trophic levels continue to be defined and studied simply on the basis of "trophic position" or "trophic guild," with no attention given to amount of energy and nutrient transfer between resources and consumers, attempts to identify causal mechanisms underlying trophic cascades will be uninformative.

So how should ecologists approach teaching about trophic cascades in lake systems? Paradigms and buzzwords can be ambiguous and even misleading[63,258], especially when based on inadequately tested hypotheses. Such unsupported concepts can be restated with such frequency that a danger emerges where speculations could be elevated to ecological laws simply over time[63,259]. As pointed out by del Solar and Marone[260], the dynamism of ecology's hypotheses and theories suggests that dogmatic teaching in this field is very likely to have negative effects on future ecology and ecologists. If textbooks include information about the trophic cascade effects of piscivorous fish in lakes, they should include information about the controversy over trophic cascades[3,6,253] along with realistic overviews of the ecological effects of piscivores on lakes (e.g., see Lampert and Sommer[261] and Wetzel[63]). Authors need to objectively review biomanipulation, including its strengths and weaknesses, and not paint an oversimplistic picture about stability of the managed systems or the low costs of the biomanipulation approaches[63,121]. Trophic cascades of piscivorous fish do exist in natural systems[262], but their role in regulating algal biomass in lakes is still relatively unstudied and its implications for lake management uncertain.

### ACKNOWLEDGEMENTS

We thank Matthew Chumchal, Weston Nowlin, and Val Smith for helpful comments and editorial suggestions.
